# A case of reflux laryngitis after iodine staining for esophageal squamous cell carcinoma

**DOI:** 10.1002/deo2.306

**Published:** 2023-10-26

**Authors:** Sho Yatsuji, Yoshitsugu Misumi, Akiko Tamiya, Kouichi Nonaka

**Affiliations:** ^1^ Department of Digestive Endoscopy Tokyo Women's Medical University Hospital Tokyo Japan; ^2^ Department of Otorhinolaryngology‐Head and Neck Surgery Tokyo Women's Medical University Hospital Tokyo Japan

**Keywords:** endoscopic submucosal dissection, esophageal cancer, iodine staining, laryngitis, Lugol's solution

## Abstract

Iodine staining allows for clear visualization of the lesion boundaries of esophageal squamous cell carcinoma and is used as the gold standard for detecting and diagnosing the extent of the cancer. Heartburn and retrosternal pain are known side effects; however, no reports of pharyngitis or laryngitis exist. Therefore, we present a case of laryngitis caused by iodine reflux. An 80‐year‐old female patient underwent endoscopic submucosal dissection for superficial esophageal cancer. During the operation, a reflux of the iodine used for diagnosing the extent of the lesion occurred, and she experienced laryngitis accompanied by hoarseness postoperatively, which improved with steroid administration. Laryngitis due to iodine reflux may cause airway stenosis, and preventing reflux requires anterograde application of iodine and spraying iodine as gently and locally as possible.

## INTRODUCTION

Endoscopic treatment of superficial esophageal cancer requires an accurate diagnosis of the extent of the lesion to achieve R0 resection. Iodine staining can more clearly visualize lesion boundaries and is preferred for the endoscopic diagnosis of esophageal cancer unless there is a special reason (e.g., iodine hypersensitivity). However, iodine is an irritant to mucous membranes, which can increase the frequency of heartburn and retrosternal pain after application in high concentrations.^1^ A few reports of esophagitis after iodine spraying are available; however, no reports of pharyngitis or laryngitis exist.^2^, ^3^ Therefore, we report a case of laryngitis with hoarseness caused by reflux of iodine solution used during esophageal endoscopic submucosal dissection (ESD).

## CASE REPORT

**FIGURE 1:**
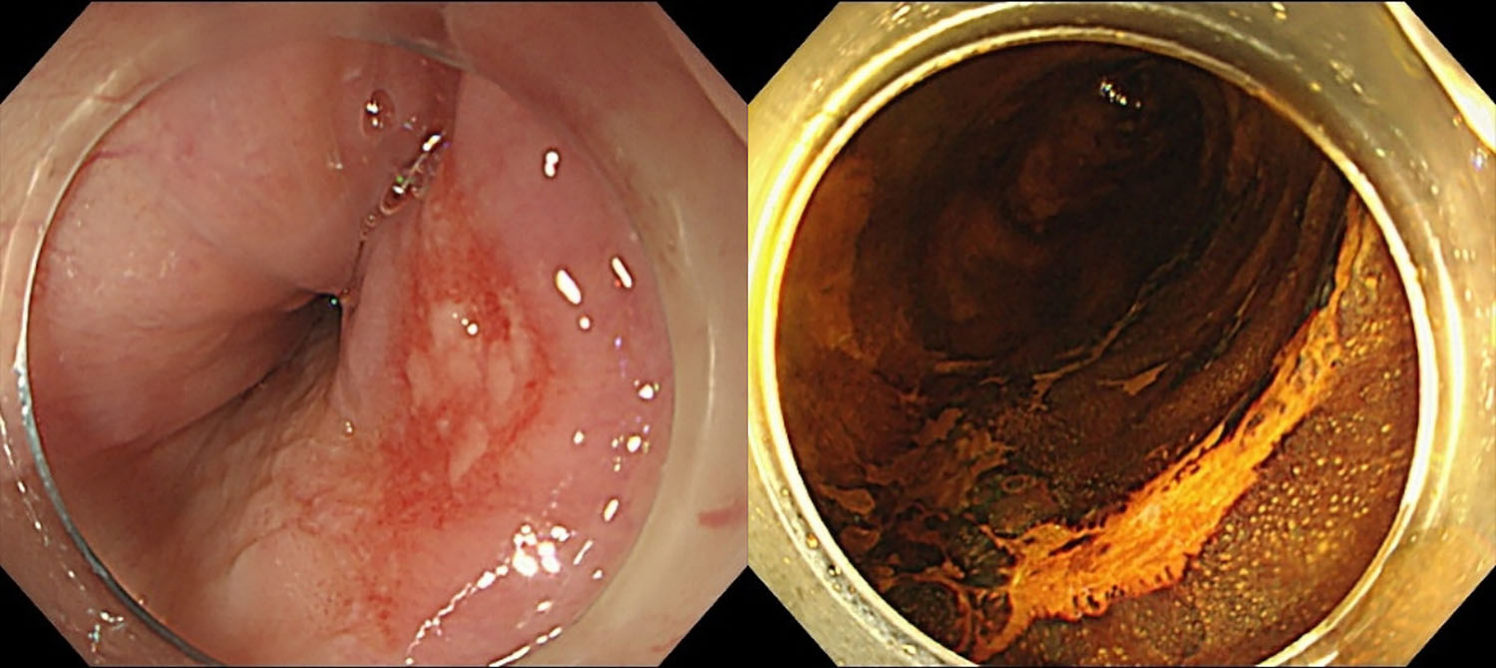
Image showing a 16‐mm type 0‐IIc tumor in the posterior wall of the esophagus at 32 cm from the incisor. The lesion was unstained on iodine staining.

**FIGURE 2:**
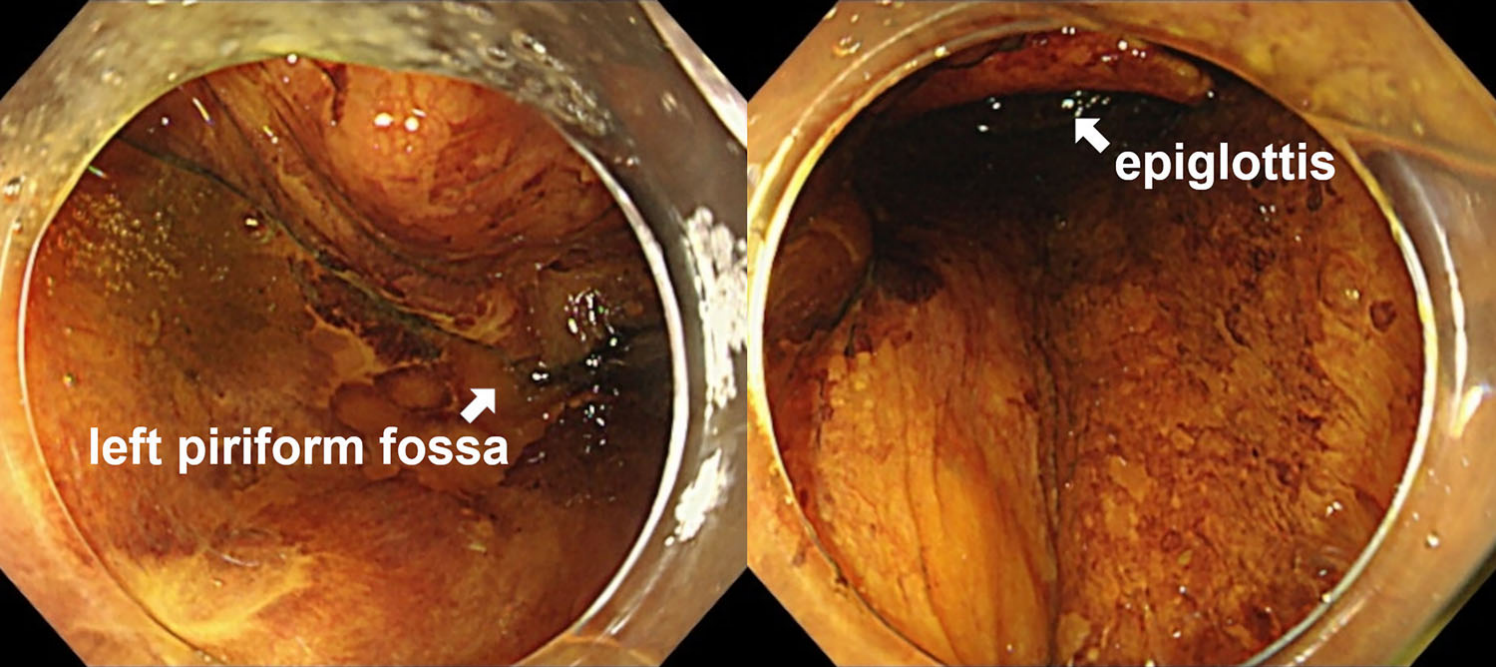
During endoscopic submucosal dissection, the pharyngeal mucosa was stained by the reflux of iodine solution into the laryngopharynx.

**FIGURE 3:**
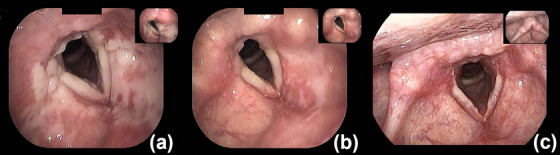
(a) Laryngoscopic image on postoperative day 6. Edema on both vocal cords and a white erosion from the false to the true vocal cords were observed. (b) Laryngoscopic image on postoperative day 9. Edema on both vocal cords improved, and the erosion from the false to the true vocal cords showed an improving tendency. (c) Laryngoscopic image on postoperative day 13. The erosion showed further improvement.

**FIGURE 4:**
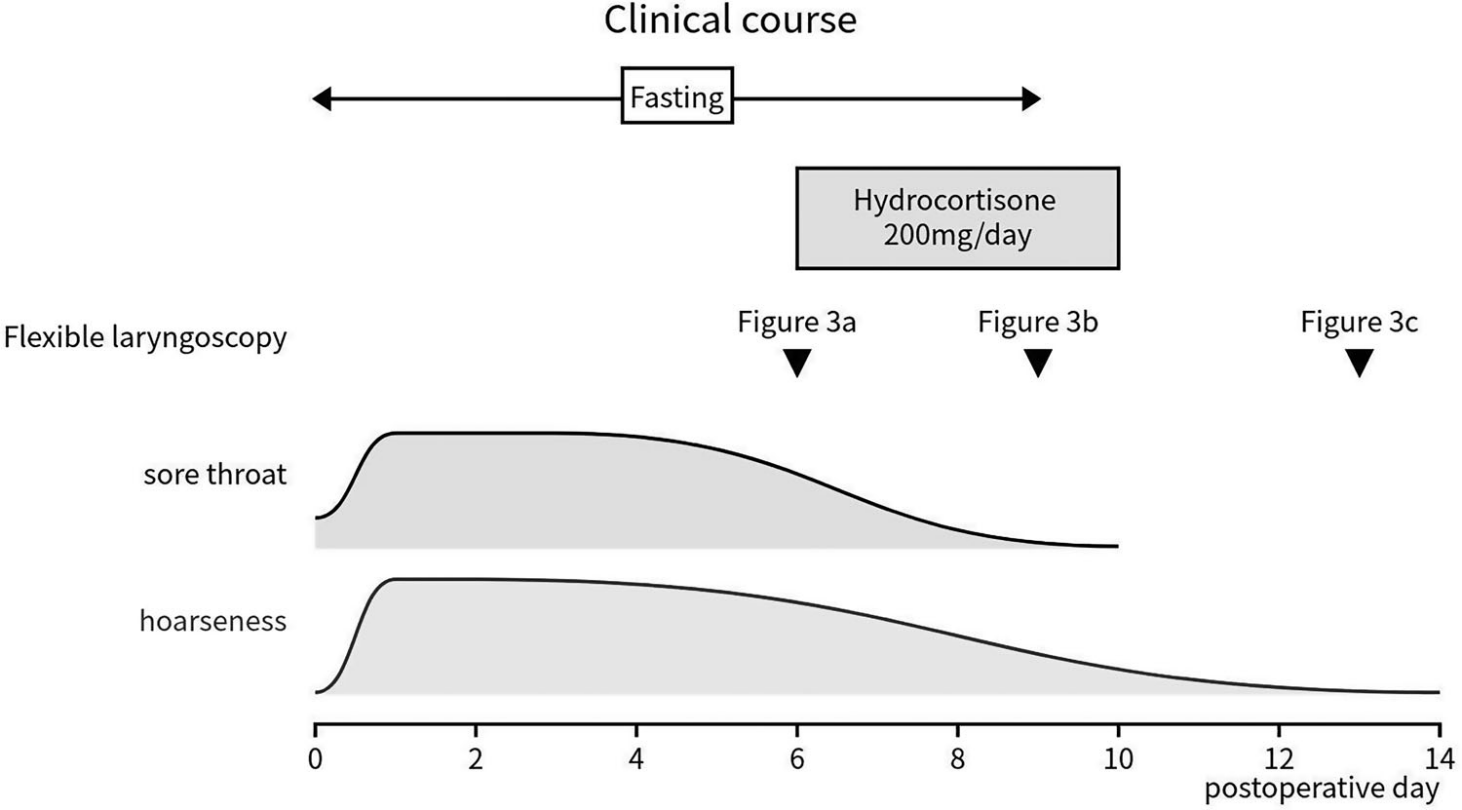
Clinical course, including symptoms and treatment, and fasting, during the hospital stay following endoscopic submucosal dissection.

An 80‐year‐old female patient with a history of ESD for esophageal cancer was hospitalized to undergo ESD for metachronous ectopic recurrent lesions. The patient has been drinking 1000 cc of beer daily for over 40 years. She smoked 40 cigarettes daily for over 40 years but quit 16 years ago. Additionally, the patient had no allergies or history of adverse effects of iodine staining during endoscopy. A 16‐mm type 0‐IIc tumor was located in the posterior wall of the esophagus at 32 cm from the incisor (Figure 1). Sedation during ESD was provided via intravenous midazolam at an initial dose of 3 mg, and carbon dioxide insufflation was used. To diagnose the extent of the lesion, 10 mL of 1.5% Lugol's solution was sprayed using an endoscopic dye spray tube (KS Jet; Kaigen Pharma Co., Ltd.), followed by iodine staining by pulling the scope from the esophagogastric junction to 25 cm of the incisors. During ESD, when removing the scope in preparation for the use of the string clip for traction, reflux of the iodine solution into the laryngopharynx and stained pharyngeal mucosa was observed (Figure 2). The solution was quickly aspirated, and the ESD procedure was continued, with the procedure completed in approximately 50 min without complications. During ESD, the patient had only a few vomiting reflexes and no choking. However, she had a sore throat from the night of the operation and hoarseness to the extent that she had no voice and experienced pain when swallowing from the day after the operation. Therefore, the patient consulted an otorhinolaryngology department and underwent flexible laryngoscopy, which revealed edema and a white erosion in the larynx and vocal cords on both sides (Figure 3a) and saliva inflow into the larynx. She was diagnosed with drug‐induced laryngitis due to iodine. No decrease in oxygen saturation level was observed, although 200 mg of hydrocortisone was administered by drip infusion from postoperative days 6–9 due to concerns about upper airway stenosis. Flexible laryngoscopy was repeated on postoperative day 9, revealing improved laryngeal edema and reduced erosion (Figure 3b). The hoarseness gradually improved, and on postoperative day 13, the erosion was further reduced (Figure 3c). Finally, the patient was discharged. During an outpatient visit on postoperative day 27, the hoarseness had disappeared, and no particular subjective symptoms were observed (Figure 4).

## DISCUSSION

This case revealed that laryngitis can be caused by iodine reflux into the laryngopharynx. Iodine staining during endoscopy is known to cause degeneration of the esophageal squamous epithelium and esophagitis.^2^, ^3^ A case of gastric mucosal injury has also been reported.^4^ However, no case report was identified on PubMed with the key terms “iodine” OR “Lugol” AND “pharyngitis” OR “laryngitis,” making ours the first case report. Iodine reportedly has a direct stimulating effect on mucous membranes.^5^, ^6^

In endoscopic laryngopharyngeal surgery, neutralizing with sodium thiosulphate and washing several times with saline after iodine staining are common practices. Compared to endoscopic laryngopharyngeal surgery, this case was not tracheally intubated and could not be neutralized or washed. Therefore, we believe that the prolonged exposure of iodine to the pharyngeal mucosa may have been a risk for severe laryngitis development.

Sore throat after iodine staining of the esophagus, hoarseness, and other symptoms appear to be indirectly related to endoscopy. However, upper airway stenosis and mucosal damage caused by severe laryngeal edema have been reported to occur due to a suicide attempt by taking diluted iodine tincture, and these symptoms should be noted when considering the possibility of iodine‐induced laryngitis.^7^

A method for reducing the side effects of iodine staining is anterograde rather than retrograde application, which results in less heartburn and retrosternal pain, less consumption of iodine solution, and less reflux of iodine solution into the upper airway and oral cavity.^8^ In this case, the reflux of iodine solution to the laryngopharynx might have been caused by vigorous or retrograde spraying. Therefore, reflux may be prevented by the anterograde application of iodine solution as gently and locally as possible. We believe that, in older adults, reflux should be of particular concern because their upper esophageal sphincter is weaker than that of young patients.^9^ Slight head elevation may be effective in preventing reflux. Furthermore, airway protection with tracheal intubation should be considered in the treatment of cervical and upper thoracic esophageal cancer. If a sore throat or hoarseness occurs after iodine staining, iodine reflux‐induced laryngitis should be differentiated without the assumption that it is caused by endoscope insertion.

## CONFLICT OF INTEREST STATEMENT

None.

## References

[1] Gotoda T , Kanzaki H , Okamoto Y *et al*. Tolerability and efficacy of the concentration of iodine solution during esophageal chromoendoscopy: A double‐blind randomized controlled trial. Gastrointest Endosc 2020; 91: 763–770. 31669091 10.1016/j.gie.2019.10.022

[2] Thuler FP , de Paulo GA , Ferrari AP . Chemical esophagitis after chromoendoscopy with Lugol's solution for esophageal cancer: Case report. Gastrointest Endosc 2004; 59: 925–926. 15173818 10.1016/s0016-5107(04)00173-7

[3] Park JM , Seok Lee I , Young Kang J *et al*. Acute esophageal and gastric injury: Complication of Lugol's solution. Scand J Gastroenterol 2007; 42: 135–137. 17190773 10.1080/00365520600825141

[4] Sreedharan A , Rembacken BJ , Rotimi O . Acute toxic gastric mucosal damage induced by Lugol's iodine spray during chromoendoscopy. Gut 2005; 54: 886–887. 15888800 10.1136/gut.2004.061739 PMC1774547

[5] Fagundes RB , de Barros SG , Pütten AC *et al*. Occult dysplasia is disclosed by Lugol chromoendoscopy in alcoholics at high risk for squamous cell carcinoma of the esophagus. Endoscopy 1999; 31: 281–285. 10376452 10.1055/s-1999-122

[6] Kondo H , Fukuda H , Ono H *et al*. Sodium thiosulfate solution spray for relief of irritation caused by Lugol's stain in chromoendoscopy. Gastrointest Endosc 2001; 53: 199–202. 11174292 10.1067/mge.2001.110730

[7] Sorimachi K , Ikegami Y , Suzuki T *et al*. Case of iodism complicated with severe airway stenosis due to pharyngolaryngeal edema. Chudoku Kenkyu 2013; 26: 305–309. Japanese. 24483010

[8] Tian X , Yang W , Chen WQ . Comparative efficacy and safety of anterograde vs. retrograde iodine staining during esophageal chromoendoscopy: A single‐center, prospective, parallel‐group, randomized, controlled, single‐blind trial. Front Med 2021; 8: 764111. 10.3389/fmed.2021.764111 PMC8655331 34901080

[9] Mei L , Dua A , Kern M *et al*. Older age reduces upper esophageal sphincter and esophageal body responses to simulated slow and ultraslow reflux events and post‐reflux residue. Gastroenterology 2018; 155: 760–770.e1. 29803837 10.1053/j.gastro.2018.05.036 PMC6120791

